# Automatic Functional Shoulder Task Identification and Sub-Task Segmentation Using Wearable Inertial Measurement Units for Frozen Shoulder Assessment

**DOI:** 10.3390/s21010106

**Published:** 2020-12-26

**Authors:** Chih-Ya Chang, Chia-Yeh Hsieh, Hsiang-Yun Huang, Yung-Tsan Wu, Liang-Cheng Chen, Chia-Tai Chan, Kai-Chun Liu

**Affiliations:** 1 Department of Physical Medicine and Rehabilitation, Tri-Service General Hospital, School of Medicine, National Defense Medical Center, Taipei 114, Taiwan; gradesboy@gm.ym.edu.tw (C.-Y.C.); crwu98@gmail.com (Y.-T.W.); clctsgh@yahoo.com.tw (L.-C.C.); 2 Department of Physical Therapy and Assistive Technology, National Yang-Ming University, Taipei 112, Taiwan; 3 Department of Biomedical Engineering, National Yang-Ming University, Taipei 112, Taiwan; g39904006@ym.edu.tw (C.-Y.H.); huangshoy@ym.edu.tw (H.-Y.H.); ctchan@ym.edu.tw (C.-T.C.); 4 Research Center for Information Technology Innovation, Academia Sinica, Taipei 115, Taiwan

**Keywords:** shoulder task identification, sub-task segmentation, frozen shoulder, wearable inertial measurement units, accelerometer, gyroscope

## Abstract

Advanced sensor technologies have been applied to support frozen shoulder assessment. Sensor-based assessment tools provide objective, continuous and quantitative information for evaluation and diagnosis. However, the current tools for assessment of functional shoulder tasks mainly rely on manual operation. It may cause several technical issues to the reliability and usability of the assessment tool, including manual bias during the recording and additional efforts for data labeling. To tackle these issues, this pilot study aims to propose an automatic functional shoulder task identification and sub-task segmentation system using inertial measurement units to provide reliable shoulder task labeling and sub-task information for clinical professionals. The proposed method combines machine learning models and rule-based modification to identify shoulder tasks and segment sub-tasks accurately. A hierarchical design is applied to enhance the efficiency and performance of the proposed approach. Nine healthy subjects and nine frozen shoulder patients are invited to perform five common shoulder tasks in the lab-based and clinical environments, respectively. The experimental results show that the proposed method can achieve 87.11% F-score for shoulder task identification, and 83.23% F-score and 427 mean absolute time errors (milliseconds) for sub-task segmentation. The proposed approach demonstrates the feasibility of the proposed method to support reliable evaluation for clinical assessment.

## 1. Introduction

Frozen shoulder (FS) is a common joint condition that causes stiffness and pain among people aged from 40 to 65 years [1], especially in women [2]. The stiffness and pain of shoulder joints lead the limitation to the range of motion in all movement planes of the shoulder joints. FS has great impacts on the quality of daily life and activities of daily living (ADL) performance [2,3]. The common treatments in FS patients involving physical therapy and joint shoulder injection aim to relieve pain, improve joint mobility, and increase the independent ability. In order to support clinical decisions, there is a requirement of objective assessment for clinical evaluations and follow-up progresses [4].

Goniometry measurements [5] and questionnaires [6] are common evaluation tools for clinical FS assessment. However, these traditional assessment approaches have several challenges and limitations related to inter-rater reliability, respondent interpretation, and cultural diversity [7,8,9]. In recent years, inertial measurement units (IMUs) have been used to develop objective evaluation systems. Joint evaluation systems using IMUs have advantages in simplification of implementation, cost, and computation complexity. They have the potential to continuously and accurately measure dynamic and static range of motion of shoulder joints, including flexion, extension and rotation [10]. Previous studies have shown the reliability of measurement systems with inertial sensors for elbow and shoulder movement in laboratory environments [10,11,12,13].

For FS patients, wearable IMUs are also implemented to objectively measure functional abilities while the questionnaires can only provide subjective scores from the patients (e.g., shoulder pain and disability index [14] and simple shoulder score [15]). These works extracted movement features and parameters to evaluate the performance of functional shoulder tasks. However, the whole measurement still relies on manual operation. For example, researchers or clinical professionals have to manually label the starting and ending time of the shoulder tasks from the continuous signals. Then, they label the spotted shoulder task with the correct task information. These additional efforts may decrease the feasibility and usability of the IMU-based evaluation systems in the clinical setting.

To tackle the aforementioned challenges, this pilot study aims to propose an automatic functional shoulder task identification and sub-task segmentation system using wearable IMUs for FS assessment. We hypothesized that the proposed wearable-based systems would be reliable and feasible to automatically provide shoulder task information for clinical evaluation and assessment. Several typical pattern recognition and signal processing techniques (e.g., symmetry-weighted moving average, sliding window and principal component analysis), machine learning models (e.g., support vector machine, k-nearest-neighbor and classification and regression tree), and rule-based modification are applied to the proposed system to accurately identify shoulder tasks and segment sub-tasks from continuous sensing signal. Moreover, a hierarchical approach is applied to enhance the reliability and efficiency of the proposed system. The novelty and contribution of this pilot study are listed as follows:

- This work firstly proposes a functional shoulder task identification system for automatic shoulder task labeling while the traditional functional measurement in clinical setting still relies on manual operation.
- The proposed approach can provide not only shoulder task information (e.g., cleaning head) but also sub-task information (e.g., lifting hands to head, washing head and putting heads down). Such sub-task information has the potential to support clinical professionals for further analysis and examination.
- The feasibility and the effectiveness of the proposed shoulder identification and sub-task segmentation is validated on nine FS patients and nine healthy subjects.

## 2. Related Works

In recent years, automatic movement identification and segmentation algorithms have been proposed to clinical evaluation and healthcare applications [16,17,18,19,20]. The main objective of identification and segmentation algorithms is to spot the starting and ending points of target activates precisely. For example, previous studies have developed diverse approaches to automatically and objectively obtain detailed lower limb and trunk movement information, such as sitting, standing, walking and turning [16]. Such reliable segmentation approaches can assist clinical professionals for various disease assessment, involving Parkinson’s disease [17], fall prediction [18] and dementia [19]. Similar approaches are also applied to upper limb assessments in stroke patients. Biswas et al. [20] proposed segmentation algorithms using a single inertial sensor to gather three basic movements from the complicated forearm activities in healthy and stroke patients, involving extension, flexion and rotation. However, few studies focus on the development of automatic systems in FS patients [11]. Most evaluation tools for FS assessment still relied on manual operation [10,21,22,23,24].

Various machine learning (ML) approaches are applied to automatically identify human movements for healthcare applications [25,26,27,28,29]. Generally, there are two categories for ML: discriminative and generative approaches. The typical discriminative approaches involving k- nearest-neighbors (k-NN) [25], classification and regression tree (CART) [26] and support vector machine (SVM) [26] aim to optimize the rules or decision boundaries to separate classes. Such approaches have shown the high- speed processing and reliable detection performance for movement segmentation. Another approach is generative models, such as hidden Markov models (HMM) [27], which are built based on probabilistic models to identify continuous movements. Generative approaches have better abilities to the more complicated activities and temporal order problems. Additionally, diverse deep learning approaches are widely applied to movement segmentation [28] and human activity recognition [29], e.g., convolutional neural networks (CNN) and recurrent neural networks (RNN). They have superior classification ability compared to traditional ML approaches. However, generative and deep learning approaches require a large dataset to ensure the performance of the detection model, while the data requirements for discriminative approaches are comparatively low.

## 3. Methods

**Figure 1:**
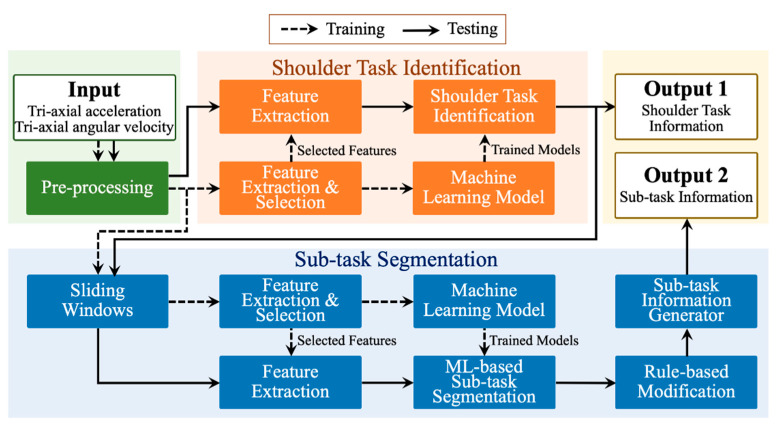
The framework of the automatic shoulder task identification and sub-task segmentation.

The framework of the proposed automatic shoulder task identification and sub-task segmentation is shown in Figure 1. The brief introduction of the whole training and testing stages for the identification and segmentation is as follows:

- Input and pre-processing: In the beginning, accelerometers and gyroscopes are utilized to collect shoulder task sequences (Input). Then, the sensing sequences are pre-processed with the moving average technique to filter the noises. These pre-processed sequences are spilt into the training set and testing set for the training and testing stages, respectively.
- Training for shoulder task identification: The feature extraction process with 12 feature types is firstly applied to the pre-processed sequences. Then, the principal component analysis is employed to reduce the size of the features and select the critical features for training machine learning models. Next, the machine learning model is trained with the selected features of the training set for shoulder task identification. Various machine learning techniques, including SVM, CART, and kNN, are investigated in this work. The parameter optimization for each technique is executed in this stage.
- Training for sub-task segmentation: First, the sliding window technique divides the pre-processed sequences into segments. Then, the feature extraction and dimension reduction techniques are employed to obtain the critical features from the segments. Lastly, the machine learning model for ML-based sub-task segmentation is built with the critical features. During the training stage, several machine learning techniques (e.g., SVM, CART, and kNN) and their optimized parameters are also explored.
- Testing for shoulder task identification: Initially, the selected features are extracted from the shoulder task sequence of the testing set. Then, these features are identified using the trained machine learning model to output the shoulder task information (output 1).
- Testing for sub-task segmentation: After the testing stage of the shoulder task identification, the sliding window technique is firstly applied to the shoulder task sequence to gather a sequence of segments. Secondly, the feature extraction process is employed to the segments to obtain selected features. Thirdly, the process of ML-based sub-task segmentation classifies these segments and the corresponding features using the trained machine learning models and outputs a sequence of the identified class labels. Fourthly, the rule-based modification is utilized to modify the output of the ML-based sub-task segmentation. Finally, the sub-task information generator generates a sequence of sub-task labels based on the classified and modified class labels and outputs it as the sub-task information (output 2).

### 3.1. Participants

Participants were outpatients at a rehabilitation department of Tri-service general hospital who were diagnosed with primary FS between June 2020 and September 2020. The patients were included if they have shoulder pain with a limited range of motion more than 3 months and age from 20 to 70 years old. Participants were diagnosed with primary FS according to standardized history, physical examination, and ultrasonographic evaluation by an experiment physiatrist. Patients were excluded if they had any of the following: full or massive thickness tear of the rotator cuff on ultrasonography or magnetic resonance imaging (MRI); secondary FS (secondary to other causes, including metabolic, rheumatic, or infectious arthritis; stroke; tumor; or fracture); and acute cervical radiculopathy.

The study was approved by the institutional review board (TSGHIRB No.: A202005024) at the university hospital, and all participants gave written informed consent. Our research procedure followed the Helsinki Declaration. All participants were assured that their participation was entirely voluntary and that they could withdraw at any time. Nine healthy adults (height: 170.6 ± 7.9 cm, weight: 75.1 ± 17.0 kg, age: 27.0 ± 5.0 years old) and nine FS patients (height: 164.3 ± 11.1 cm, weight: 66.3 ± 14.4 kg, age: 56.4 ± 9.9 years old) participated in the experiments.

### 3.2. Experimental Protocol and Data Collection

**Figure 2:**
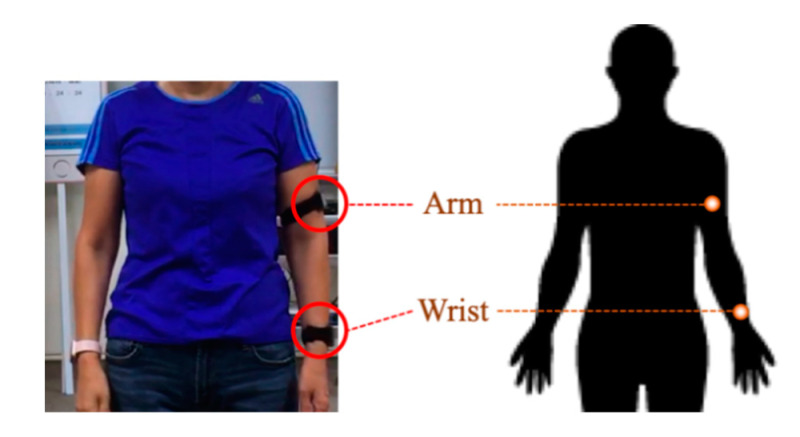
An illustration of the sensor placements.

Two IMUs placed on the arm and wrist are employed to sense the upper limb movement, as shown in Figure 2. Similar sensor placements have been selected in previous works [20,21]. The sensors placed on the arm and wrist can catch information of upper limb movement while performing shoulder tasks. The used IMU (APDM Inc., Portland, OR, USA) involves a tri-axial accelerometer, tri-axial gyroscope, and tri-axial magnetometer. In this study, only the tri-axial accelerometer (range: ±16 g; resolution: 14 bits) and tri-axial gyroscope (range: ±2000°/s; resolution: 16 bits) work for the data. The data is collected with a sampling frequency of 128 Hz.

The experiment is executed in the lab-based and clinical environments for healthy and FS subjects, respectively. Each subject is asked to perform five shoulder tasks once, including cleaning head, cleaning upper back and shoulder, cleaning lower back, placing an object on a high shelf, and putting/removing an object from the back pocket. These shoulder tasks have been widely adopted for shoulder function assessment and evaluation in previous works [21,22]. The performed shoulder tasks and the corresponding three sub-tasks are listed in Table 1. Each task consists of three sub-tasks. Totally, there are 90 shoulder task sequences (18 subjects × 5 shoulder tasks). The participants are free to execute tasks in their ways with basic manual instruction. The sub-tasks are performed continuously within the same shoulder task. Mean sub-task time performed by healthy and FS patients is listed in Table 2.

The external camera synchronized with inertial sensors is applied to provide reference information for the ground truth labeling, including starting and ending points of shoulder tasks. During the experiment, the camera is put in front of the subjects. The frame per second of the camera is 30 Hz.

### 3.3. Data Pre-Processing

This study applies the symmetry-weighted moving average (SWMA) technique to the sensing signals to reduce the noise and artifacts for shoulder task identification and segmentation. This pre-processing technique has been applied to other applications while the sensors are placed on the upper limbs, including eating activity recognition and daily activity recognition [30,31]. SWMA technique determines different weights to sample points within the determined ranges. The data points closer to the central point are assigned with higher weights.

Suppose the sensing data of any shoulder task sequence is defined as $S=\left\{s_{i}|i=1,\;2,\ldots ,n_{R}\right\}$, where $n_{R}$ is the total number of the data samples from the sequence. The pre-processed sensing data point ${\tilde{s}}_{t}$ at time t with the determined range m is defined as follows:

(1) $${\tilde{s}}_{t}=\frac{1}{Total_{\delta }}\left(\delta_{0}s_{i}+\left(\sum_{i=1}^{\frac{m+1}{2}-1}\delta_{i}\left(s_{t+i}+s_{t-i}\right)\right)\right),$$

(2) $$Total_{\delta }=\sum_{i=0}^{\frac{m+1}{2}-1}\delta_{i},$$

where $Total_{\delta }$ is the sum of all determined weights, $\delta_{0}$ is $\frac{m+1}{2}$ and $\delta_{i}=\left\{\delta_{o}-i|i=1,\;2,\ldots ,\delta_{0}\right\}$. For example, if m is 5, $s_{3}^{\prime }=\left[\left(s_{3}\times 3+s_{2}\times 1+s_{4}\times 1+s_{1}\times 0.5+s_{5}\times 0.5\right)/\left(3+1+1+0.5+0.5\right)\right]$. The SWMA with m=9 is applied to this study.

### 3.4. Shoulder Task Identification

#### 3.4.1. Feature Extraction

The main objective of the feature extraction process is to extract movement characteristics from the continuous sensing data for shoulder task identification. There are two feature categories that have been applied to catch motion features, such as statistical and kinematic features. The common statistical features involving mean, standard deviation (StD), variance (var), maximum (max), minimum (min), range, kurtosis, skewness, and correlation coefficient (CorrCoef) have been applied to the field of activity recognition applications [32]. These nine statistical features are applied to this work. Also, kinematic features have been applied to upper limb movement recognition systems in several clinical applications, such as stroke rehabilitation and assessment [33]. This study employs three general kinematic features, such as the number of velocity peaks (NVP), zero crossing (NZR), and mean crossing (NMR) for shoulder task identification.

Suppose a sequence of data from a sensor is defined as $\tilde{S}=\left\{{\tilde{s}}_{i}|i=1,\;2,\ldots ,n_{R}\right\}$, where $n_{R}$ is the total number of the data samples from the sequence. Any sample point $s_{i}$ includes data collected from a tri-axial sensor ${\tilde{s}}_{i}=\left\{{\tilde{r}}_{x_{i}},\;{\tilde{r}}_{y_{i}},{\tilde{r}}_{z_{i}}\right\}$. Then, the feature extraction process is applied to the shoulder sequence. The utilized features are listed in Table 3. 

In this work, the sensing data of the shoulder task sequence from two IMUs is defined as ${\tilde{S}}_{seq}=\left\{{\tilde{s}}_{i}|i=1,\;2,\ldots ,n_{seq}\right\}$, where $n_{seq}$ is the total number of ${\tilde{S}}_{seq}$. Any sample point ${\tilde{s}}_{i}$ of ${\tilde{S}}_{seq}$ is defined as:

$${\tilde{S}}_{i}=\left\{{\tilde{a}}_{xi}^{wrist},{\tilde{a}}_{yi}^{wrist},{\tilde{a}}_{zi}^{wrist},{\tilde{g}}_{xi}^{wrist},{\tilde{g}}_{yi}^{wrist},{\tilde{g}}_{zi}^{wrist},{\tilde{a}}_{xi}^{arm},{\tilde{a}}_{yi}^{arm},{\tilde{a}}_{zi}^{arm},{\tilde{g}}_{xi}^{arm},{\tilde{g}}_{yi}^{arm},{\tilde{g}}_{zi}^{arm}\right\}$$

**Figure 3:**
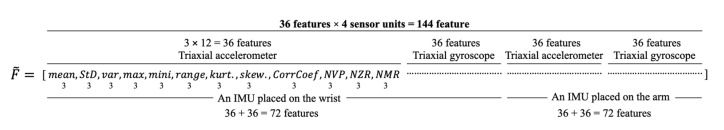
A formation of the extracted features from the IMUs.

The formation of the extracted features from ${\tilde{S}}_{seq}$ is show in Figure 3. There are two IMUs, four sensors (2 accelerometers + 2 gyroscopes), and a total of 144 features (4 sensor units × 36 features) are obtained.

#### 3.4.2. Feature Selection

During the training stage, the feature selection process is applied to all extracted features after the feature extraction. This is because the size of all features (144 features) is quite big for the systems.

Using a suitable feature selection technique can simplify the computing processes, which is beneficial for training and testing stages. This study utilizes principal component analysis (PCA) [34] to select critical features and reduce the number of features in dealing with multi-dimensional time sequence data. PCA aims to find a linear transformation matrix that transforms the raw feature vectors $\tilde{F}=\left[{\tilde{f}}_{1},{\tilde{f}}_{2},\ldots ,{\tilde{f}}_{k}\right]$ to lower dimensional feature vectors $\hat{F}=\left[{\hat{f}}_{1},{\hat{f}}_{2},\ldots ,{\hat{f}}_{l}\right]$, where k=144 is the number of the raw feature vectors and l is the number of the transformed feature vectors.

Firstly, the covariance matrix $C_{f}$ is calculated based on the variance maximization of the projected data. Then, the eigenvalues $\lambda =\left(\lambda_{1},\lambda_{2},\ldots ,\lambda_{k}\right)$ and eigenvectors $\nu =\left(\nu_{1},\nu_{2},\ldots ,\nu_{k}\right)$ can be determined based on $C_{f}$. Note that the eigenvectors ν are the principal components, where that first eigenvector has the largest variance.

In the dimension reduction process, the l eigenvectors with the most explained components are kept, where l≤k. A threshold thres=0.99 is set to keep 99% variance information of the raw feature vectors. The minimum value of l is determined as Equation (3):

(3) ∑i=1lλi∑i=1kλi≥thres

For the shoulder task identification, the number of features is reduced from 144 to 35 after PCA and dimension reduction processes. Compared to the original raw feature vectors, the system using the transformed feature sets has the potential to reduce computational complexity for the classification of the shoulder task.

#### 3.4.3. Shoulder Task Identification Using Machine Learning

Suppose there is a set of class labels $C=\left(c^{1},c^{2},\ldots ,c^{n_{c}}\right)$, where $n_{C}$ is the number of the class labels. The training set $\Gamma^{train}=\left\{\left({\hat{F}}_{i}^{train},c_{i}\right)|i=1,\;2,\ldots ,n_{train}\right\}$ has $n_{train}$ pairs of feature vectors ${\hat{F}}_{i}^{train}$ and the corresponding label $c_{i}$. In the training stage, the machine learning technique can optimize the parameters θ of a classification model by minimizing the classification loss on $\Gamma^{train}$. For the shoulder task identification, $n_{C}=5$ is the number of the shoulder tasks.

In the testing stage, given that the testing set $\Gamma^{test}=\left\{{\hat{F}}_{i}^{test}|i=1,\;2,\ldots ,n_{test}\right\}$ has $n_{test}$ feature vectors. Each ${\hat{F}}_{i}^{test}$ is mapped to a set of class labels C with the corresponding confidence score $P_{i}=\left\{p_{i}^{j}|j=1,\;2,\ldots ,n_{C}\right\}$ using the trained classification model Η with the optimized parameters θ:

(4) $$p_{i}\left(c|{\hat{F}}_{i}^{test},\theta \right)=Η\left({\hat{F}}_{i}^{test},\theta \right),$$

where c∈C. Then, we select the class label with the maximum confidence score as the final classification output:

(5) $$c_{i}=\underset{c\in C,\;\;\;p\in P_{i}}{argmax}p\left(c|{\hat{F}}_{i}^{test},\theta \right).$$

There are various machine learning models have been applied to segment human movements and recognize activities in other clinical applications [16,17,18,19,20]. At this moment, several machine learning techniques requiring a lot of data volume for model training are not considered in this work, involving HMM, CNN, and RNN. Therefore, we focus on exploring the feasibility of the following machine learning models for shoulder task identification:

- Support vector machine (SVM): The main objective of the SVM model is to find a hyperplane to separate two classes. It maximizes the margin between two classes to support distinct classification with more confidence. Since the number of the classes are more than two, we employ one-vs-all techniques to multi-class classification with a radial basis kernel function.
- K-nearest-neighbors (kNN): kNN approach is also called as a lazy classifier as this approach actually does not require any training process. The main idea of this approach is to determine the class of the testing data based on the major voting of nearest k neighbors. The determination of the value k is application-dependent, which have critical influences on the performance of the classifier. In this work, a range of k from 1 to 9 is explored. The results show that k=7 achieves the best detection performance.
- Classification and regression tree (CART): The CART approach is a binary tree that can tackle classification and regression problems. The branch size and the process of the splitting is determined by measure of the Gini impurity. This approach has advantages in easy implementation and high processing speed.

The feasibility and reliability of the explored techniques have been validated in the field of activity recognition [29].

### 3.5. Sub-Task Segmentation

#### 3.5.1. Sliding Window

There are several windowing approaches that have been proposed to divide the continuous data into chunks [35], involving sliding window, event-defined window and activity-defined window techniques. This work uses the sliding window to segment the data into small segments. This windowing approach is very popular in the field of activity recognition due to its simple realization and fast processing speed.

**Figure 4:**
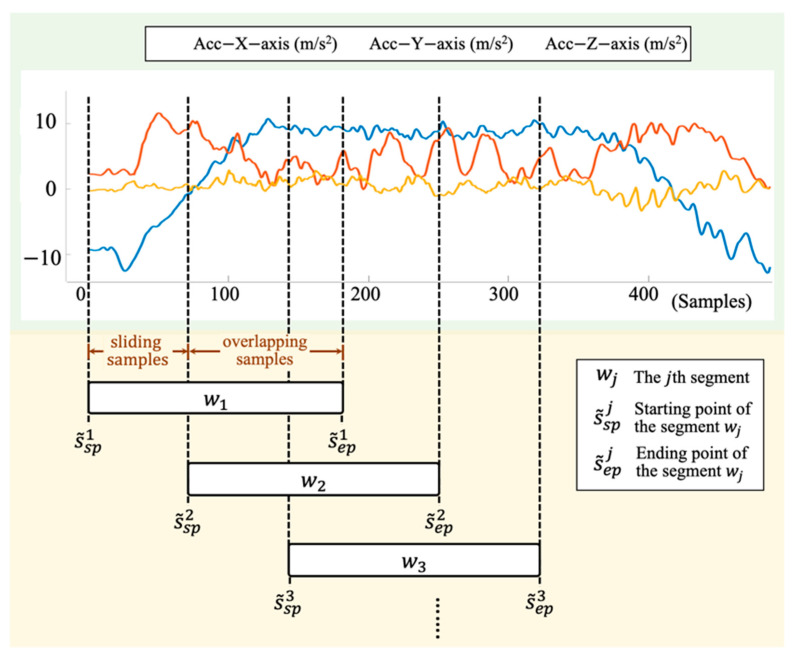
An illustration of the sliding window on the sensing signals. ${\tilde{s}}_{sp}^{j}$ and ${\tilde{s}}_{ep}^{j}$ are the starting and ending points of the segment $w_{j}$, where j=1,2,3,… . The sliding samples is the distances from ${\tilde{s}}_{sp}^{j}$ to ${\tilde{s}}_{sp}^{j+1}$. The overlapping samples is the number of the overlapping data samples between segments $w_{j}$ and $w_{j+1}$.

Suppose the pre-processed sensing data of the shoulder task sequence from two IMUs is defined as ${\tilde{S}}_{seq}=\left\{{\tilde{s}}_{i}|i=1,\;2,\ldots ,n_{seq}\right\}$, where $n_{seq}$ is the total number of ${\tilde{S}}_{seq}$. The sliding window technique is applied to ${\tilde{S}}_{seq}$ with several parameters, including window size ws, the starting point of the segment sp, ending point of the segment ep, sliding samples ss. The pseudocode of the sliding window is described in Algorithm 1 and illustrated in Figure 4.


**Algorithm 1:** Sliding Window.
**Input:**
the pre-processed sensing data ${\tilde{S}}_{seq}=\left\{{\tilde{s}}_{i}|i=1,2,\ldots ,n_{seq}\;\right\}$**,** window size ws, the starting point of the segment sp, ending point of the segment ep, sliding samples ss
**Output:**
a set of segments $W=\left\{w_{j}|j=1,2,\ldots ,n_{sl}\;\right\}$1:
***Begin***
2:**initialize**sp←1, ep←ws and j←13:**while** e$p\leq n_{seq}$ do4:
$\;\;\;\;\;\;\;\;\;\;w_{j}\leftarrow \left\{{\tilde{s}}_{sp}^{j},{\tilde{s}}_{sp+1}^{j},\ldots ,{\tilde{s}}_{ep-1}^{j},{\tilde{s}}_{ep}^{j}\right\}$
5:
$\;\;\;\;\;\;\;\;\;\;\;\;\;\;\;\;\;\;W\leftarrow w_{j}$
6:
                 j←j+1
7:
                sp←sp+ss
8:ep←sp+ws−19:
**endwhile**
10:
***End***



After the process of sliding window, a set of segments obtained from the shoulder task sequences ${\tilde{S}}_{seq}$ is defined as $W=\left\{w_{j}|j=1,\;2,\ldots ,n_{sl}\right\}$, where $n_{sl}$ is the total number of segments obtained from ${\tilde{S}}_{seq}$. Any segment is defined as $w_{j}=\left\{{\tilde{s}}_{sp}^{j},{\tilde{s}}_{sp+1}^{j},\ldots ,{\tilde{s}}_{ep-1}^{j},{\tilde{s}}_{ep}^{j}\right\}$, where ${\tilde{s}}_{sp}$ and ${\tilde{s}}_{ep}$ are the starting and ending points of the segment. Note that op is defined as the overlapping percentage between $w_{j}$ and $w_{j+1}$, where $j+1\leq n_{sl}$. op can be calculated as follows:

(6) $$op=\frac{os}{ws},$$

(7) os=ws−ss,

where os is the overlapped samples.

The window size has great impact on the system performance while using the sliding window technique. A range of window sizes from 0.1 to 1.5 s with a fixed overlapping of 50% is tested to explore the reliability of the proposed automatic sub-task segmentation.

#### 3.5.2. Training Stage for Sub-Task Segmentation

Given that there is a set of segments $W_{TrSet}=\left\{w_{j}^{train}|j=1,\;2,\ldots ,n_{TrSet}\right\}$ obtained from the pre-processed shoulder task sequences using sliding window, where $w_{j}^{train}=\left\{{\tilde{s}}_{j}^{train}|j=1,\;2,\ldots ,n_{ws}\right\}$ contains $n_{ws}$ sample points. Any ${\tilde{s}}_{i}^{train}$ containing the sensing data collected from the wrist and arm is defined as ${\tilde{S}}_{i}^{train}=\left\{{\tilde{a}}_{xj}^{wrist},{\tilde{a}}_{yj}^{wrist},{\tilde{a}}_{zj}^{wrist},{\tilde{g}}_{xj}^{wrist},{\tilde{g}}_{yj}^{wrist},{\tilde{g}}_{zj}^{wrist},{\tilde{a}}_{xj}^{arm},{\tilde{a}}_{yj}^{arm},{\tilde{a}}_{zj}^{arm},{\tilde{g}}_{xj}^{arm},{\tilde{g}}_{yj}^{arm},{\tilde{g}}_{zj}^{arm}\right\}$. The training process is as follows:

- Firstly, $w_{TrSet}$ are initially extracted with nine types of statistical features and three types of kinematic features to obtain training feature vectors ${\tilde{U}}^{train}=\left\{{\tilde{F}}_{j}^{train}|j=1,\;2,\ldots n_{TrSet}\right\}$, where ${\tilde{F}}_{j}^{train}=\left\{{\tilde{f}}_{j,1}^{train},{\tilde{f}}_{j,2}^{train},\ldots ,{\tilde{f}}_{j,k}^{train}\right\}$ and k=144.
- Then, PCA is also applied to ${\tilde{U}}^{train}\;to$ obtain dimensionless feature vectors ${\hat{U}}^{train}=\left\{{\hat{F}}_{j}^{train}|j=1,\;2,\ldots n_{TrSet}\right\}$, where ${\hat{F}}_{j}^{train}=\left\{{\hat{f}}_{j,1}^{train},{\hat{f}}_{j,2}^{train},\ldots ,{\hat{f}}_{j,\overset{´}{l}}^{train}\right\}$ and $\overset{´}{l}\leq k$. In this paper, the size of the utilized feature vectors $\overset{´}{l}$ for different windows is reduced from 144 to less than 50.
- After the processes of feature extraction and selection, a training set ${\overset{´}{\Gamma }}^{train}=\left\{\left({\hat{F}}_{j}^{train},c_{j}^{train}\right)|i=1,\;2,\ldots ,n_{TrSet}\right\}$ is created, where $n_{TrSet}$ is the number of feature vectors, and $c_{i}^{train}$ is the corresponding label of ${\hat{F}}_{i}^{train}.$ In this work, there is a set of class labels $\overset{´}{C}=\left({\overset{´}{c}}^{1},{\overset{´}{c}}^{2},\ldots ,{\overset{´}{c}}^{n_{\overset{´}{c}}}\right)$, where $n_{\overset{´}{C}}$ is 3, including sub-task A, B, and C.
- Finally, using a machine learning technique learns the parameters $\overset{´}{\theta }$ of the machine learning model $\overset{´}{Η}$ from ${\overset{´}{\Gamma }}^{train}$. Several typical ML approaches are also explored for sub-task segmentation, such as SVM, CART, and kNN.

#### 3.5.3. Testing Stage for Sub-Task Segmentation Using Machine Learning Models, Rule-Based Modification and Sub-Task Information Generator

There are three main processes for sub-task segmentation: ML-based identification, rule-based modification and sub-task information generator. The first process is to employ ML approaches to segment and identify sub-tasks. Several typical machine learning approaches are tested, such as SVM, CART, and kNN. However, mis-segmentation and mis-identification is unavoidable during the process. Therefore, the second process is to correct the errors from the ML-based approach. The modification process modifies fragmentation errors as the identified results are irrational to the context. For example, a continuous data stream identified as sub-task B “washing head” should not involve other sub-tasks (e.g., lifting hands or putting hands down). Finally, the generator generates the sub-task information based on the outputs of the rule-based modification.

**Figure 5:**
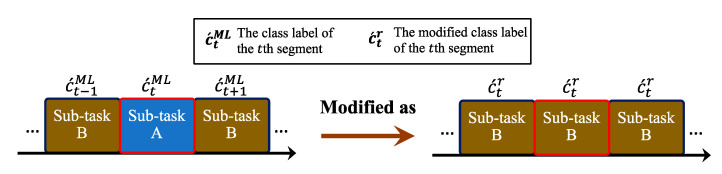
An illustration of the modification process to the fragmentation errors. The “sub-task A” of ${\overset{´}{c}}_{t}$ is the misidentified result that is modified as “sub-task B” according to the proposed rule-based modification.

**Figure 6:**
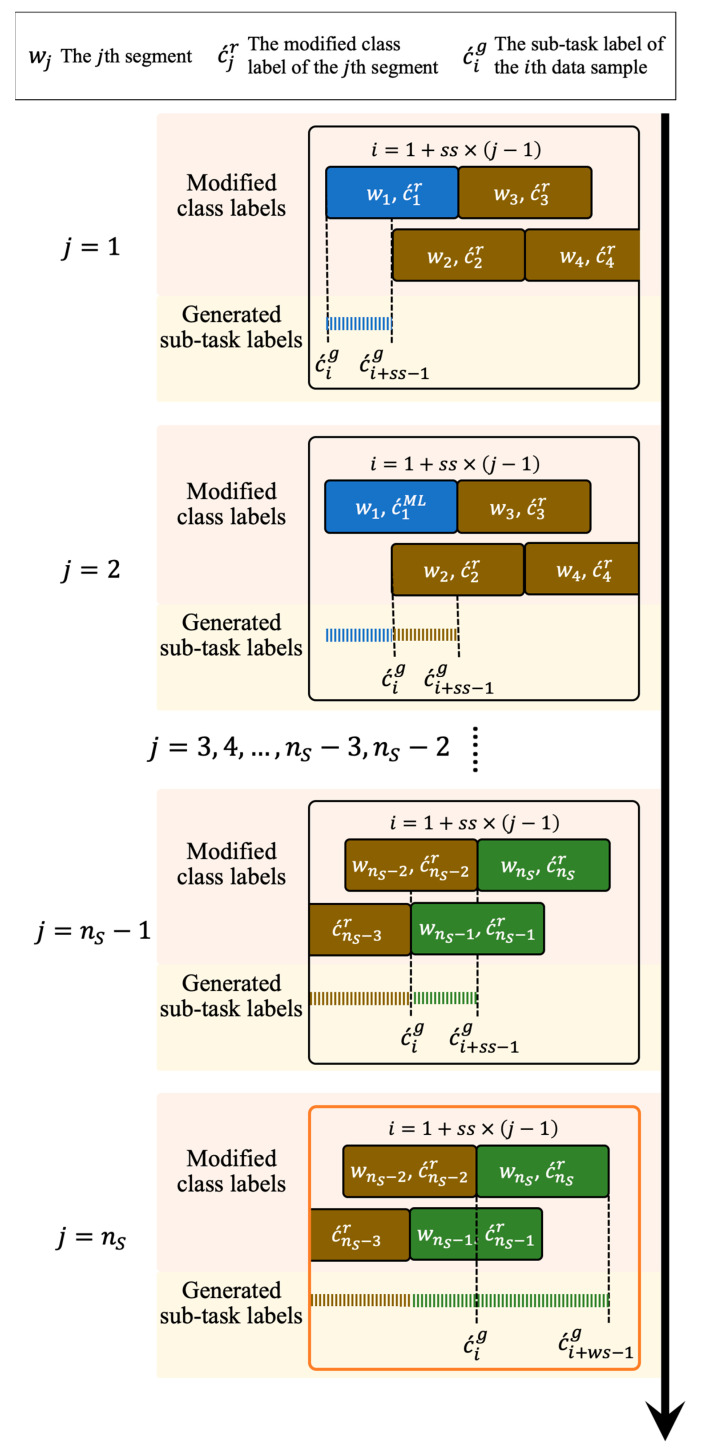
An illustration of the sub-task information generator. A sequence of sub-task labels $D_{g}=\left\{{\overset{´}{c}}_{i}^{g}|i=1,\;2,\ldots ,n_{g}\right\}$ is obtained based on a sequence of modified class labels $D_{r}=\left\{{\overset{´}{c}}_{j}^{r}|j=1,\;2,\ldots ,n_{s}\right\}$. For the first $n_{s}-1$ modified class labels, each ${\overset{´}{c}}_{j}^{r}$ maps to a sequence of sub-task labels {${\overset{´}{c}}_{i}^{g}$,$\;{\overset{´}{c}}_{i+1}^{g}$,…,$\;{\overset{´}{c}}_{i+ss-2}^{g},{\overset{´}{c}}_{i+ss-1}^{g}$ }, where i=1+ss×(j−1). Finally, a sequence of sub-task labels {${\overset{´}{c}}_{i}^{g}$,$\;{\overset{´}{c}}_{i+1}^{g}$,…,$\;{\overset{´}{c}}_{i+ws-2}^{g},{\overset{´}{c}}_{i+ws-1}^{g}$ } is obtained from the last modified class label ${\overset{´}{c}}_{n_{s}}^{r}$.

Given that a set of segments $W_{TeSet}=\left\{w_{1}^{test},w_{2}^{test},\ldots ,w_{n_{S}}^{test}\right\}$ and the corresponding feature vectors ${\hat{U}}^{test}=\left\{{\hat{F}}_{1}^{test},{\hat{F}}_{2}^{test},\ldots ,{\hat{F}}_{n_{S}}^{test}\right\}$ are obtained from a pre-processed shoulder task sequence of the testing set ${\tilde{S}}_{TeSeq}=\left\{{\tilde{s}}_{1},{\tilde{s}}_{2},\ldots ,{\tilde{s}}_{n_{TeSeq}}\right\}$ by using the sliding window technique and feature extraction with the selected features, where $n_{TeSeq}$ and $n_{S}$ are the total number of ${\tilde{S}}_{TeSeq}$ and $W_{TeSet}$, respectively. The detailed ML-based sub-task segmentation and rule-based modification processes in the testing stage is described as follows:

- Firstly, the mapped confidence score ${\overset{´}{P}}_{i}=\left\{{\overset{´}{p}}_{i}^{1},\;{\overset{´}{p}}_{i}^{2},\ldots ,{\overset{´}{p}}_{i}^{n_{\overset{´}{c}}}\right\}$ of a set of class labels $\overset{´}{C}=\left({\overset{´}{c}}^{1},{\overset{´}{c}}^{2},\ldots ,{\overset{´}{c}}^{n_{\overset{´}{c}}}\right)$ from each ${\hat{F}}_{i}^{test}$ is calculated, where $n_{\overset{´}{c}}$ is the total number of $\overset{´}{C}$.
- Secondly, each ${\hat{F}}_{i}^{test}$ maps to a class label ${\overset{´}{c}}^{ML}$ with the maximum confidence score by using the trained machine learning model $\overset{´}{Η}$ and the optimized parameters $\overset{´}{\theta }$. A sequence of classified class labels $D_{ML}=\left\{{\overset{´}{c}}_{1}^{ML},{\overset{´}{c}}_{2}^{ML},\ldots ,{\overset{´}{c}}_{n_{S}}^{ML}\right\}$ is generated from ${\hat{U}}^{test}$ using $\overset{´}{Η}$ and $\overset{´}{\theta }.$
- Thirdly, the rule-based modification is applied to $D_{ML}$ to obtain a sequence of modified class labels $D_{r}=\left\{{\overset{´}{c}}_{1}^{r},{\overset{´}{c}}_{2}^{r},\ldots ,{\overset{´}{c}}_{n_{S}}^{r}\right\}$. If ${\overset{´}{c}}_{t}^{ML}$ is different from ${\overset{´}{c}}_{t-1}^{ML}$ and ${\overset{´}{c}}_{t+1}^{ML}$, and ${\overset{´}{c}}_{t-1}^{ML}$ is equal to ${\overset{´}{c}}_{t+1}^{ML}$ then ${\overset{´}{c}}_{t}^{ML}$ would be modified as the sub-task of ${\overset{´}{c}}_{t-1}^{ML}$ and ${\overset{´}{c}}_{t+1}^{ML}$, where ${\overset{´}{c}}_{t}^{ML}\in D_{ML}$ and $2\leq t\leq n_{S}-1$. An example to illustrate the modification process is shown in Figure 5.
- Finally, a generator generates a sequence of sub-task labels $D_{g}=\left\{{\overset{´}{c}}_{1}^{g},{\overset{´}{c}}_{2}^{g},\ldots ,{\overset{´}{c}}_{n_{g}}^{g}\right\}$ based on $D_{r}$, where $n_{g}$ is the total number of $D_{g}$ and determined as:
  (8) $$n_{g}=ws+ss\times \left(n_{S}-1\right),$$
  where ws and ss are window size and sliding samples, respectively. The processes of the sub-task information generator are illustrated in Figure 6 and the corresponding pseudocode is shown in Algorithm 2.

**Algorithm 2:** Sub-task Information Generator.
**Input:**
a sequence of modified class labels $D_{r}=\left\{{\overset{´}{c}}_{j}^{r}|j=1,2,\ldots ,n_{s}\right\}$, window size ws, sliding samples ss
**Output:**
a sequence of sub-task labels $D_{g}=\left\{{\overset{´}{c}}_{i}^{g}|i=1,2,\ldots ,n_{g}\right\}$1:
***Begin***
2:
**  initialize**
*i*
←1
3:**  for** j=1 **to** $n_{s}-1$ **do** //for the first $n_{s}-1$ modified class labels4:
**   while **
i≤(j×ss)
5:
$\;\;\;\;{\overset{´}{c}}_{i}^{g}\leftarrow {\overset{´}{c}}_{j}^{r}$
6:
$\;\;\;\;D_{g}\leftarrow {\overset{´}{c}}_{i}^{g}$
7:
    i←i+1
8:
**   endwhile**
9:
** endfor**
10: **for** i **to** i+ws **do** //for the last $n_{s}$ modified class labels11:
$\;\;\;\;{\overset{´}{c}}_{i}^{g}\leftarrow {\overset{´}{c}}_{ns}^{r}$
12:
$\;\;\;\;D_{g}\leftarrow {\overset{´}{c}}_{i}^{g}$
13:
** endfor**
14:
***End***



### 3.6. Performance Evaluation and Statistical Analysis

The whole system implementation and statistical analysis are done using the Statistics and Machine Learning Toolbox in Matlab 2017b (MathWorks Inc., Natick, MA, USA).

This study utilizes a leave-one-subject-out cross-validation approach [32] to validate the system performance of the proposed shoulder task identification and sub-task segmentation. This validation approach divides the dataset into k folds based on the subjects, where k is the number of subjects; one fold is kept as the testing set and the remaining k-1 folds are utilized for the training. The whole process repeats k times until each fold is used as the testing set. Finally, the system outputs the average results of k folds.

In order to evaluate the reliability of the shoulder task identification, several typical metrics are utilized for performance evaluation, including sensitivity, precision and F-score [36] as shown in Equations (9)–(11):

(9) $$\mathrm{sensitivity}=\frac{\mathrm{TP}}{\mathrm{TP}\;+\;\mathrm{FN}}$$

(10) $$\mathrm{precision}=\frac{\mathrm{TP}}{\mathrm{TP}\;+\;\mathrm{FP}}$$

(11) $$F-\mathrm{score}=2\times \frac{\mathrm{sensitivity}\;\times \;\mathrm{precision}}{\mathrm{sensitivity}\;+\;\mathrm{precision}}$$

where TP, FP, TN, and FN are true positive, false positive, true negative, and false negative. F-Score is the harmonic mean of precision and recall, which is a common approach to evaluate the reliability and performance of classification systems.

**Figure 7:**
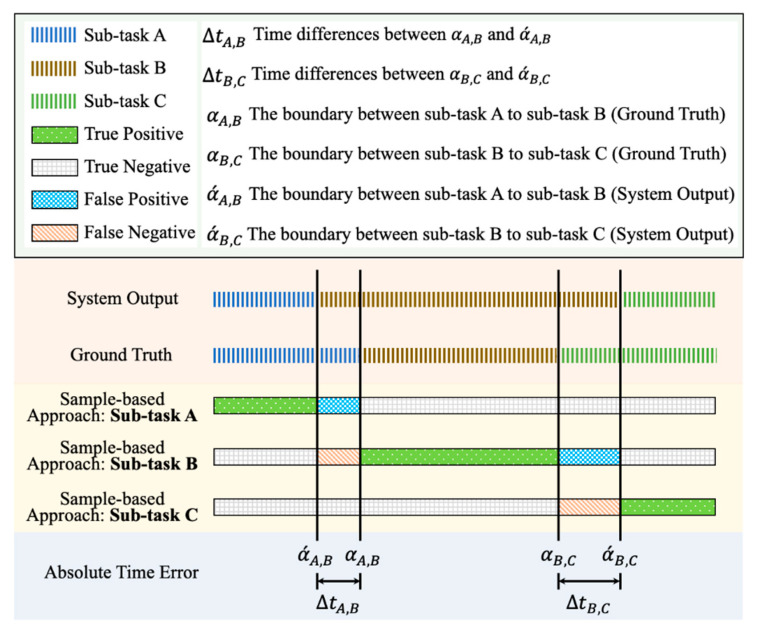
An illustration of the annotation for the performance evaluation of sub-task segmentation, including true positive, true negative, false positive, false negative, and absolute time error.

There are two evaluation and analysis approaches applied for the evaluation of sub-task segmentation: the sample-based approach [36] and mean absolute time errors (MATE) [37,38,39]. An illustration of the evaluation approaches for sub-task segmentation is shown in Figure 7. The first one is to calculate the number of TP, FP, TN, and FN based on the sample-by-sample mapping between the ground truth and system outputs. Then, the sensitivity, precision and F-score are applied to assess the system reliability based on the mapping results. The second approach is to calculate the average of the absolute time errors between the reference and identified boundaries, where the boundary is the edge between two sub-tasks. There are two MATE values calculated for the proposed sub-task segmentation:

- ${\mathrm{MATE}}_{A,B}$: MATE of the boundaries between sub-task A and sub-task B.
- ${\mathrm{MATE}}_{B,C}$: MATE of the boundaries between sub-task B and sub-task C.
- ${\mathrm{MATE}}_{\mathrm{overall}}$: MATE of all boundaries between sub-task A and sub-task B and between sub-task B sub-task C.

## 4. Results

The experimental results of the shoulder task identification are shown in Table 4. The results show that the shoulder task identification using SVM model can achieve 87.06% sensitivity, 88.43% precision and 87.11% F-score, and outperform that using other ML models. However, the proposed approach using SVM model is still weak to tackle several shoulder tasks such as T3 (cleaning lower back) and T5 (putting/removing an object in/form the back pocket) while the F-score of other shoulder tasks can achieve over 90%.

The sensitivity, precision, and F-score of the sub-task segmentation using different ML approaches and window sizes are presented in Table 5, Table 6 and Table 7, respectively. Generally, the sub-task segmentation using SVM and kNN models have the similar performance in sensitivity, precision, and F-score, which outperforms that using kNN model. The experimental results show that the proposed segmentation approach with SVM model can achieve the best overall performance in sensitivity (82.27%), precision (85.07%) and F-score (83.23%) while the worst performance is with CART model. Furthermore, using SVM model has the best F-score of 86.53%, 82.75%, and 82.42% in the sub-task A, sub-task B, and sub-task C, respectively.

The results also reveal that the F-score of the sub-task segmentation model using SVM and kNN models significantly decreases when the window is larger than 1.0 s. Most of them achieve the best performance as window sizes are 0.2 and 0.3 s. However, the performance using CART model achieves the best F-score with the window size of 1.5 s.

Table 8, Table 9 and Table 10 presents the sub-task segmentation performance of ${\mathrm{MATE}}_{A,B}$, ${\mathrm{MATE}}_{B,C}$ and ${\mathrm{MATE}}_{\mathrm{overall}}$ using different machine learning models and window sizes for all subjects, healthy subject and FS patients, respectively. Overall, the proposed segmentation using kNN achieves the lowest ${\mathrm{MATE}}_{A,B}$, ${\mathrm{MATE}}_{B,C}$ and ${\mathrm{MATE}}_{\mathrm{overall}}$ in most subject groups. However, the best machine learning models for ${\mathrm{MATE}}_{\mathrm{overall}}$ and ${\mathrm{MATE}}_{B,C}$ of FS patients are SVM and CART, respectively. The lowest ${\mathrm{MATE}}_{\mathrm{overall}}$ of all subjects, healthy subjects and FS patients are 427, 273, and 517 ms, respectively. Also, the experimental results reveal that the MATE of healthy subjects is lower than that of the FS patients.

The impact of window sizes in the sub-task segmentation performance of ${\mathrm{MATE}}_{A,B}$, ${\mathrm{MATE}}_{B,C}$ and ${\mathrm{MATE}}_{\mathrm{overall}}$ is similar to that of sensitivity, precision and F-score. The proposed segmentation approach with different machine learning models have the lowest MATE values when the window size is smaller or equal to 1.0 s. Particularly, the results show that the proposed segmentation system using window sizes of 0.1 and 1.0 s can achieve the lowest ${\mathrm{MATE}}_{A,B}$, ${\mathrm{MATE}}_{B,C}$ and ${\mathrm{MATE}}_{\mathrm{overall}}$.

**Figure 8:**
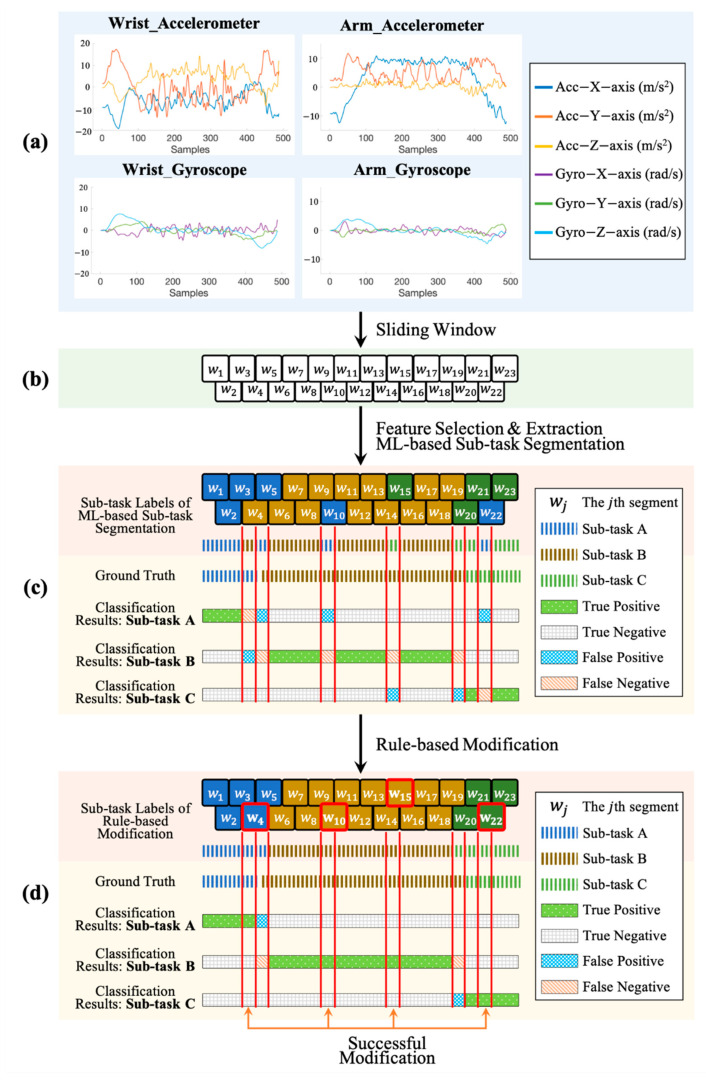
An example of the signal performed by the health subject and the processes of the proposed sub-task segmentation. (**a**) The accelerometer and gyroscope signals collected from the IMUs placed on the wrist and arm. (**b**) The divided segments obtained from the process of sliding window. In this example, there are 23 segments (**c**) The classification results for sub-task A, B and C after the processes of feature extraction and ML-based sub-task segmentation, where the TP, TN, FP, and FN are annotated. (**d**) The classification results after the processes of rule-based modification, where the modified sliding segments are highlighted in red square (e.g., $w_{4}$, $w_{10}$, $w_{15}$, $w_{22}$) and the successful modification results are annotated.

An example to demonstrate the processes of ML-based identification and rule-based modification for sub-task segmentation on the healthy subject is shown in Figure 8. It presents that a complete segment is often divided into fragments when the system used ML-based segmentation only, as shown in Figure 8c. For example, a segment of sub-task B is divided into 4 fragments. The proposed rule-based modification can correct the segmentation errors caused by ML-based sub-task segmentation, as presented in Figure 8d. After the processes of ML-based sub-task segmentation and rule-based modification, the segmentation errors of this work mainly occur in the boundaries between two sub-tasks, which decrease the performance of the proposed sub-task segmentation approach.

## 5. Discussion

Various sensor technologies have been applied to develop objective evaluation systems, including range of motion measurement and function evaluation. To tackle the issues in labeling errors and bias during the measurement, we propose an automatic functional shoulder task identification and sub-task segmentation system using wearable IMUs for FS assessment. The proposed approach can achieve 87.11% F-score for shoulder task identification, and 83.23% F-score, 387 ${\mathrm{MATE}}_{A,B}$ and 403 ${\mathrm{MATE}}_{B,C}$ for sub-task segmentation. The proposed system has the potential to support clinical professionals in automatic shoulder task labeling and sub-task information obtainment.

The results show that the proposed shoulder task identification has poor performance on T3 and T5 as the F-score on them are lower than 80%. This is because several FS patients are unable to move hands to the lower back but they can reach the back pocket while performing T3. The execution of T3 and T5 performed by the patients have very similar movement patterns. Such a situation confuses the models for identification of T3 and T5, even for SVM model.

Several machine learning models have been applied in this work, including SVM, CART and kNN. Previous works have shown the feasibility and the effectiveness of these models in movement identification and segmentation [16,17,18,19,20]. The proposed segmentation approach using SVM and kNN models can achieve the best performance in F-score and MATE, respectively. However, the differences between their segmentation performance are very close in the two evaluation performance approaches. Considering that the kNN model has the advantages of less computation complexity and simple implementation, the kNN model is more suitable for the proposed system.

**Figure 9:**
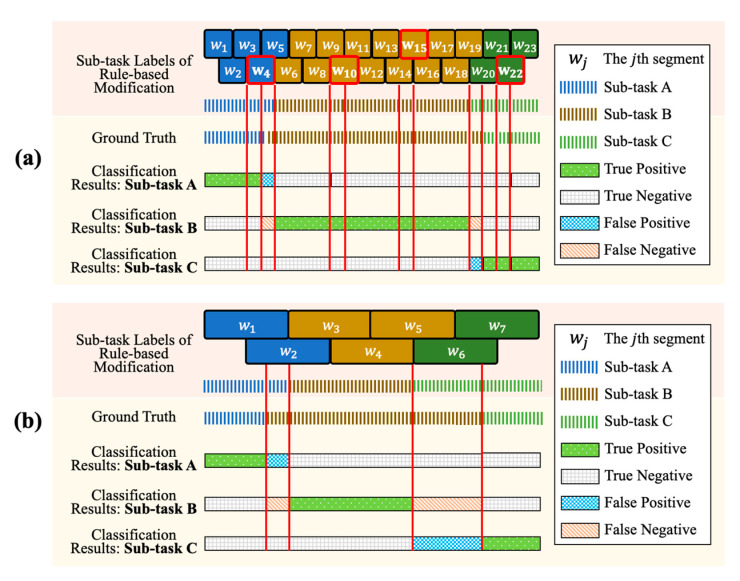
An illustration of the segmentation performance using smaller and larger window sizes. (**a**) The classification results using smaller window size, where the window size is 0.5 s. (**b**) The classification results using larger window size, where the window size is 1.5 s.

Previous studies have shown that the sliding window approach is sensitive to the window sizes [35]. The proposed sub-task segmentation approach has similar experimental results as the segmentation performance with different window sizes ranges over 10%. This is because the larger sizes of the window may smooth the movement characteristics that confuse the identification models and lead to misidentification. Also, using too larger window sizes may lead to early or late segmentation of the sub-tasks, which increases the segmentation errors of the proposed system. An illustration of the segmentation performance using smaller and larger window sizes is shown in Figure 9.

**Figure 10:**
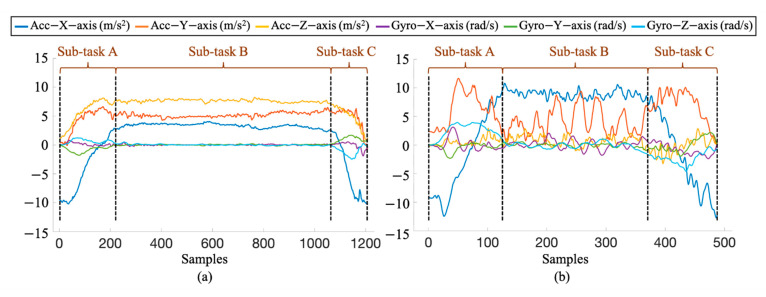
An example of the data of the T2 task “cleaning upper back and shoulder” collected from the wrist-worn sensor, which are performed by (**a**) the FS patient and (**b**)the healthy subject.

Figure 10 shows the signal of T2 “clean upper back and shoulder task” collected from the FS patient and healthy subject using a wrist-worn sensor. Due to stiffness and pain of the shoulder, the FS patients perform the shoulder task slowly and carefully with a limited range of motion. Obviously, the movement patterns of the three sub-tasks performed by the FS patient are significantly different from those performed by the healthy subject. It means the shoulder task can be performed in diverse ways according to the health status and the function of the shoulder, which leads to identification challenges of variability and similarity to the shoulder task identification and sub-task segmentation [32].

To our best knowledge, this is the first study aiming to identify and segment upper limb movements of shoulder tasks using machine learning approaches in FS patients, especially for FS assessment. Machine learning models have been successfully applied to automatic movement identification and recognition models to analyze lower limb movements in other clinical applications [16,17,18,19,20]. However, most IMU-based shoulder function assessment systems still rely on manual operation [10,21,22,23,24]. Our results demonstrate the feasibility and effectiveness of the ML-based functional shoulder task identification for supporting clinical assessment and proof of concept. Moreover, the proposed system can obtain sub-task information from continuous signals, which has the potential for further analysis and investigation of functional performance.

Some technical challenges still limit the performance of the proposed system to shoulder task identification and sub-task segmentation, including gesture time, variability, similarity, and boundary decision. We plan to test other powerful machine learning models to improve identification and segmentation performance, such as CNN, LSTM, longest common subsequence (LCSS) dynamic time warping (DTW), hidden Markov model (HMM) and conditional random field (CRF). Another limitation is that the proposed automatic system is validated on five shoulder tasks only. More shoulder tasks from other clinical tests and questionnaires are going to be explored for validation of the proposed system, e.g., simple and shoulder score [14], American Shoulder and Elbow Surgeons score [40], and so on. Furthermore, there are only nine FS patients and nine healthy subjects participating in this work. More FS patients with different functional disabilities, the different ages of healthy subjects and different disease groups will be recruited for validation and investigation.

## 6. Conclusions

In order to support FS assessment in the clinical setting, we propose a functional shoulder task identification system using IMUs for shoulder task identification and sub-task segmentation. We use several typical pattern recognition techniques, machine learning models and rule-based modification to automatically identify five shoulder tasks and segment three sub-tasks. The feasibility and reliability of this study are validated with healthy and FS subjects. The experimental results show that the proposed system has the potential to provide automatic labeling of the shoulder task and sub-task information for clinical professionals.

## Figures and Tables

**Table 1:** A list of shoulder task and sub-task.

Task ID	Shoulder Task	Sub-Task A	Sub-Task B	Sub-Task C
T1	Cleaning head	Lifting hands toward head	Washing head	Putting hands down
T2	Cleaning upper back and shoulder	Lifting hands toward upper back and shoulder	Washing upper back and shoulder	Putting hands down
T3	Cleaning lower back	Lifting hands towards lower back	Washing lower back	Putting hands down
T4	Placing an object on a high shelf	Lifting the object toward the shelf	Holding the hands on the shelf for few seconds	Putting hands down
T5	Putting/Removing an object into/from the back pocket	Putting an object into the back pocket	Holding the hands in the back pocket for few seconds	Removing an object from the back pocket

**Table 2:** Mean sub-task time performed by healthy and FS patients (s).

		Healthy Subjects	FS Patients	All Subjects
	Sub-task A	0.86 ± 0.15	1.6 ± 0.73	1.22 ± 0.62
T1	Sub-task B	3.13 ± 1.18	5.66 ± 2	4.46 ± 2.08
	Sub-task C	0.9 ± 0.2	1.18 ± 0.16	1.04 ± 0.22
	Sub-task A	1.16 ± 0.25	1.82 ± 0.85	1.45 ± 0.68
T2	Sub-task B	2.58 ± 0.75	7.81 ± 3.75	4.93 ± 3.63
	Sub-task C	1.18 ± 0.2	1.35 ± 0.21	1.29 ± 0.24
	Sub-task A	0.78 ± 0.12	1.1 ± 0.44	0.92 ± 0.35
T3	Sub-task B	3.09 ± 1.15	6.32 ± 4.19	4.94 ± 3.57
	Sub-task C	0.99 ± 0.21	0.94 ± 0.25	0.95 ± 0.24
	Sub-task A	1.53 ± 0.35	2.02 ± 0.65	1.81 ± 0.59
T4	Sub-task B	0.97 ± 0.49	1.98 ± 0.53	1.45 ± 0.7
	Sub-task C	1.52 ± 0.41	1.25 ± 0.45	1.45 ± 0.49
	Sub-task A	1.47 ± 0.46	1.79 ± 0.75	1.61 ± 0.62
T5	Sub-task B	0.9 ± 0.65	0.89 ± 0.68	0.93 ± 0.66
	Sub-task C	2.42 ± 2.44	1.37 ± 0.35	1.88 ± 1.82

**Table 3:** A list of statistical and kinematic feature types from a single sensor.

No.	Description
${\tilde{f}}_{1}$−${\tilde{f}}_{3}$	Mean of ${\tilde{r}}_{x_{i}},\;{\tilde{r}}_{y_{i}},{\tilde{r}}_{z_{i}}$
${\tilde{f}}_{4}-{\tilde{f}}_{6}$	Standard Deviation of ${\tilde{r}}_{x_{i}},\;{\tilde{r}}_{y_{i}},{\tilde{r}}_{z_{i}}$
${\tilde{f}}_{7}-{\tilde{f}}_{9}$	Variance of ${\tilde{r}}_{x_{i}},\;{\tilde{r}}_{y_{i}},{\tilde{r}}_{z_{i}}$
${\tilde{f}}_{10}-{\tilde{f}}_{12}$	Maximum of ${\tilde{r}}_{x_{i}},\;{\tilde{r}}_{y_{i}},{\tilde{r}}_{z_{i}}$
${\tilde{f}}_{13}-{\tilde{f}}_{15}$	Minimum of ${\tilde{r}}_{x_{i}},\;{\tilde{r}}_{y_{i}},{\tilde{r}}_{z_{i}}$
${\tilde{f}}_{16}-{\tilde{f}}_{18}$	Range of ${\tilde{r}}_{x_{i}},\;{\tilde{r}}_{y_{i}},{\tilde{r}}_{z_{i}}$
${\tilde{f}}_{19}-{\tilde{f}}_{21}$	Kurtosis of ${\tilde{r}}_{x_{i}},\;{\tilde{r}}_{y_{i}},{\tilde{r}}_{z_{i}}$
${\tilde{f}}_{22}-{\tilde{f}}_{24}$	Skewness of ${\tilde{r}}_{x_{i}},\;{\tilde{r}}_{y_{i}},{\tilde{r}}_{z_{i}}$
${\tilde{f}}_{25}-{\tilde{f}}_{27}$	Correlation coefficient between each pair of ${\tilde{r}}_{x_{i}},\;{\tilde{r}}_{y_{i}},{\tilde{r}}_{z_{i}}$
${\tilde{f}}_{28}-{\tilde{f}}_{30}$	Number of velocity peaks of ${\tilde{r}}_{x_{i}},\;{\tilde{r}}_{y_{i}},{\tilde{r}}_{z_{i}}$
${\tilde{f}}_{31}-{\tilde{f}}_{33}$	Number of zero crossing of ${\tilde{r}}_{x_{i}},\;{\tilde{r}}_{y_{i}},{\tilde{r}}_{z_{i}}$
${\tilde{f}}_{34}-{\tilde{f}}_{36}$	Number of mean crossing of ${\tilde{r}}_{x_{i}},\;{\tilde{r}}_{y_{i}},{\tilde{r}}_{z_{i}}$

Note. ${\tilde{r}}_{x_{i}},\;{\tilde{r}}_{y_{i}},{\tilde{r}}_{z_{i}}$ are the sample points of *x*-axis, *y*-axis and *z*-axis collected from a tri-axial sensor node.

**Table 4:** The results of the shoulder task identification using machine learning approaches (%).

Shoulder Task	Sensitivity			Precision			F-Score		
	SVM	kNN	CART	SVM	kNN	CART	SVM	kNN	CART
T1	94.12	82.35	70.59	100.00	60.87	66.67	96.97	70.00	68.57
T2	100.00	64.71	64.71	85.00	91.67	78.57	91.89	75.86	70.97
T3	88.24	76.47	82.35	71.43	86.67	77.78	78.95	81.25	80.00
T4	82.35	82.35	76.47	100.00	82.35	68.42	90.32	82.35	72.22
T5	70.59	88.24	76.47	85.71	83.33	81.25	77.42	85.71	78.79
**Overall**	87.06	78.82	74.12	88.43	80.98	74.54	87.11	79.04	74.11

Note. SVM: support vector machine; kNN: k-nearest-neighbors; CART: classification and regression tree.

**Table 5:** The sensitivity of the sub-task segmentation using machine learning approaches (%) vs. different window sizes (s).

Window Size (s)	Sub-Task A			Sub-Task B			Sub-Task C			Overall		
	SVM	kNN	CART	SVM	kNN	CART	SVM	kNN	CART	SVM	kNN	CART
0.1	77.94	**83.88**	**94.39**	90.15	87.43	64.05	78.71	61.40	48.32	**82.27 ^a^**	77.57	68.92
0.2	**83.02**	81.46	88.80	88.81	87.12	59.81	74.86	71.39	70.81	82.23	79.99	73.14
0.3	75.91	82.31	83.56	87.80	83.98	63.09	79.43	76.27	75.28	81.05	**80.85**	73.98
0.4	73.20	78.04	81.73	83.38	76.33	50.92	**83.28**	80.60	78.64	79.96	78.32	70.43
0.5	74.06	79.61	80.57	87.45	80.94	56.92	82.46	79.50	80.61	81.32	80.02	72.70
0.6	71.50	76.34	73.70	86.27	79.30	56.13	82.21	**80.63**	**81.91**	79.99	78.76	70.58
0.7	73.73	74.64	80.16	89.66	84.46	68.15	81.91	77.65	72.33	81.77	78.92	73.55
0.8	67.50	69.98	70.97	86.13	84.87	66.27	80.80	78.07	80.48	78.14	77.64	72.57
0.9	65.00	71.34	76.22	90.39	79.71	68.87	75.12	74.79	74.00	76.84	75.28	73.03
1	66.06	70.52	71.26	87.87	80.20	75.29	80.28	72.54	75.70	78.07	74.42	**74.08**
1.1	66.61	68.77	77.65	89.71	84.96	69.35	70.61	65.60	67.97	75.64	73.11	71.66
1.2	66.60	68.41	78.80	86.41	77.80	75.31	75.00	67.45	61.82	76.00	71.22	71.98
1.3	66.55	66.70	73.10	90.06	83.40	73.81	71.30	65.82	60.81	75.97	71.97	69.24
1.4	67.85	65.25	67.34	**94.35**	**91.58**	**83.01**	56.94	54.26	56.01	73.05	70.36	68.79
1.5	69.86	66.66	72.79	92.04	90.40	79.48	60.41	55.80	54.91	74.10	70.95	69.06

Note. The best performance of the column is highlighted in bold; SVM: support vector machine; kNN: k-nearest-neighbors; CART: classification and regression tree. ^a^: The best overall performance.

**Table 6:** The precision of the sub-task segmentation using machine learning approaches (%) vs. different window sizes (s).

Window Size (s)	Sub-Task A			Sub-Task B			Sub-Task C			Overall		
	SVM	kNN	CART	SVM	kNN	CART	SVM	kNN	CART	SVM	kNN	CART
0.1	93.73	81.36	57.94	**77.67**	73.22	76.97	82.96	**93.72**	76.26	84.79	82.77	70.39
0.2	90.46	85.71	71.35	78.57	76.26	77.50	86.20	90.94	56.59	**85.07 ^a^**	**84.30**	68.48
0.3	91.06	89.23	78.28	76.19	76.15	74.43	80.97	82.08	54.42	82.74	82.49	69.04
0.4	90.48	89.31	78.94	75.40	76.31	75.85	76.56	69.19	49.65	80.81	78.27	68.14
0.5	90.84	88.66	78.10	76.26	76.28	75.07	79.80	77.83	53.19	82.30	80.92	68.78
0.6	89.38	85.14	81.02	75.25	**76.38**	72.54	76.72	74.21	49.97	80.45	78.58	67.85
0.7	91.47	87.51	69.72	76.86	74.81	**77.99**	83.53	86.00	63.00	83.96	82.77	70.24
0.8	93.06	87.90	81.66	73.02	74.23	73.87	78.08	84.29	55.61	81.38	82.14	70.38
0.9	94.73	86.09	78.86	71.55	72.70	71.50	86.60	77.96	61.21	84.29	78.92	70.52
1	92.49	86.30	**83.49**	73.85	70.40	72.37	80.43	81.05	65.88	82.26	79.25	73.91
1.1	93.86	88.22	73.83	70.83	69.17	71.31	79.98	81.50	63.31	81.55	79.63	69.49
1.2	92.25	78.63	76.47	71.40	69.19	74.57	79.12	79.68	73.10	80.92	75.83	74.71
1.3	96.16	83.56	72.32	70.93	68.92	70.42	83.35	79.85	65.18	83.48	77.44	69.31
1.4	**96.93**	**91.79**	75.98	68.75	67.01	67.34	**89.40**	89.02	**84.14**	85.03	82.61	**75.82**
1.5	96.29	87.87	75.82	69.34	67.43	68.91	86.31	88.45	80.72	83.98	81.25	75.15

Note. The best performance of the column is highlighted in bold; SVM: support vector machine; kNN: k-nearest-neighbors; CART: classification and regression tree. ^a^: The best overall performance.

**Table 7:** The F-score of the sub-task segmentation using machine learning approaches (%) vs. different window sizes (s).

Window Size (s)	Sub-Task A			Sub-Task B			Sub-Task C			Overall		
	SVM	kNN	CART	SVM	kNN	CART	SVM	kNN	CART	SVM	kNN	CART
0.1	84.79	82.29	81.18	**82.75**	78.81	74.03	80.30	73.45	71.20	82.61	78.18	75.47
0.2	**86.53**	83.08	79.54	82.42	**81.06**	68.96	80.74	79.86	65.33	**83.23** ^a^	**81.33**	71.27
0.3	82.68	**85.54**	83.23	80.77	79.56	73.23	79.20	78.90	70.14	80.88	**81.33**	75.53
0.4	80.68	83.19	80.65	78.51	75.92	59.56	78.68	73.00	62.32	79.29	77.37	67.51
0.5	81.06	83.84	81.93	80.73	78.22	65.73	80.13	77.74	64.57	80.64	79.93	70.74
0.6	78.96	80.42	**83.87**	79.11	77.38	67.14	78.59	76.27	64.64	78.89	78.02	71.89
0.7	81.34	80.45	82.24	82.14	78.99	73.95	**82.42**	**80.83**	69.49	81.97	80.09	75.23
0.8	78.32	77.83	81.38	78.46	78.96	73.85	78.69	80.44	69.54	78.49	79.08	74.92
0.9	77.06	77.57	79.20	78.83	75.84	68.40	79.93	74.87	60.36	78.61	76.09	69.32
1	76.84	77.51	78.47	79.64	74.79	73.31	80.10	76.34	70.03	78.86	76.21	73.94
1.1	77.96	77.02	77.03	77.81	75.45	70.92	74.53	72.05	69.66	76.77	74.84	72.53
1.2	77.25	72.54	76.57	77.33	73.00	75.76	76.48	71.92	**72.43**	77.02	72.49	74.92
1.3	78.60	74.11	74.79	78.38	74.47	74.32	76.62	70.94	71.03	77.87	73.17	73.38
1.4	78.87	75.94	75.05	77.78	75.85	74.10	69.26	65.57	68.55	75.30	72.45	72.57
1.5	80.40	75.39	78.28	77.11	75.46	**76.67**	69.18	64.63	71.73	75.57	71.83	**75.56**

Note. The best performance of the column is highlighted in bold; SVM: support vector machine; kNN: k-nearest-neighbors; CART: classification and regression tree. ^a^: The best overall performance.

**Table 8:** The ${\mathrm{MATE}}_{A,B}$, ${\mathrm{MATE}}_{B,C}$ and ${\mathrm{MATE}}_{\mathrm{overall}}$ of all subject using different machine learning models vs. difference window sizes (s).

Window Size (sec)	$MATE_{A,B}\;(\mathrm{ms})$			$MATE_{B,C}\;(\mathrm{ms})$			$MATE_{overall}\;(\mathrm{ms})$		
	SVM	kNN	CART	SVM	kNN	CART	SVM	kNN	CART
0.1	393	**387** ^a^	569	496	466	819	445	**427** ^c^	694
0.2	**392**	438	590	472	473	1238	**433**	456	914
0.3	468	502	481	522	422	959	495	462	720
0.4	505	525	538	567	577	1549	536	551	1044
0.5	489	559	488	514	447	1419	502	503	954
0.6	536	554	**439**	555	404	1379	546	479	909
0.7	478	586	496	**430**	406	1017	454	496	757
0.8	543	551	495	554	403	1024	549	477	760
0.9	560	555	544	499	411	1425	530	483	985
1.0	556	527	558	500	**403** ^b^	909	528	465	734
1.1	537	579	612	624	515	916	581	547	764
1.2	561	581	616	591	541	739	576	561	**678**
1.3	533	494	691	564	550	723	549	522	707
1.4	523	490	676	679	560	816	601	525	746
1.5	498	497	594	698	599	**665**	598	548	630

Note. The best performance of the column is highlighted in bold; SVM: support vector machine; kNN: k-nearest-neighbors; CART: classification and regression tree. ^a^: The lowest MATE value of ${\mathrm{MATE}}_{A,B}$; ^b^: The lowest MATE value of ${\mathrm{MATE}}_{B,C}$; ^c^: The lowest MATE value of ${\mathrm{MATE}}_{\mathrm{overall}}$.

**Table 9:** The ${\mathrm{MATE}}_{A,B}$, ${\mathrm{MATE}}_{B,C}$ and ${\mathrm{MATE}}_{\mathrm{overall}}$ of healthy subject using different machine learning models vs. difference window sizes (s).

Window Size (sec)	$MATE_{A,B}\;(\mathrm{ms})$			$MATE_{B,C}\;(\mathrm{ms})$			$MATE_{overall}\;(\mathrm{ms})$		
	SVM	kNN	CART	SVM	kNN	CART	SVM	kNN	CART
0.1	**250**	**223** ^a^	458	328	397	**389**	**289**	310	**424**
0.2	314	282	450	431	301	501	373	292	476
0.3	304	274	442	365	271	514	335	**273** ^c^	478
0.4	384	311	438	470	388	796	427	350	617
0.5	325	301	426	342	316	1026	334	309	726
0.6	359	321	**359**	**281**	**267** ^b^	544	320	294	452
0.7	371	355	486	353	341	481	362	348	484
0.8	436	343	427	490	313	655	463	328	541
0.9	474	361	415	410	332	756	442	347	586
1.0	458	381	398	357	353	635	408	367	517
1.1	427	409	592	573	474	653	500	442	623
1.2	433	380	568	586	574	608	510	477	588
1.3	424	299	695	538	562	786	481	431	741
1.4	411	328	676	778	609	703	595	469	690
1.5	423	357	556	812	633	804	618	495	680

Note. The best performance of the column is highlighted in bold; SVM: support vector machine; kNN: k-nearest-neighbors; CART: classification and regression tree. ^a^: The lowest MATE value of ${\mathrm{MATE}}_{A,B}$; ^b^: The lowest MATE value of ${\mathrm{MATE}}_{B,C}$; ^c^: The lowest MATE value of ${\mathrm{MATE}}_{\mathrm{overall}}$.

**Table 10:** The ${\mathrm{MATE}}_{A,B}$, ${\mathrm{MATE}}_{B,C}$ and ${\mathrm{MATE}}_{\mathrm{overall}}$ of FS patients using different machine learning models vs. difference window sizes (s).

Window Size (s)	$MATE_{A,B}\;(\mathrm{ms})$			$MATE_{B,C}\;(\mathrm{ms})$			$MATE_{\mathrm{overall}}\;(\mathrm{ms})$		
	SVM	kNN	CART	SVM	kNN	CART	SVM	kNN	CART
0.1	**535**	**551** ^a^	680	617	536	1250	576	**544**	965
0.2	472	594	729	562	646	1975	**517** ^c^	620	1352
0.3	631	730	520	679	574	1404	655	652	962
0.4	626	739	638	664	767	2302	645	753	1470
0.5	653	817	549	686	578	1812	670	698	1181
0.6	714	788	520	830	541	2215	772	665	1368
0.7	585	817	**505**	**506**	470	1554	546	644	1030
0.8	650	759	563	618	494	1394	634	627	979
0.9	646	748	673	588	491	2094	617	620	1384
1.0	654	673	718	643	**453** ^b^	1184	649	563	951
1.1	646	749	631	675	557	1178	661	653	905
1.2	689	783	665	596	507	870	643	645	768
1.3	642	690	687	589	538	660	616	614	674
1.4	635	652	675	580	510	929	608	581	802
1.5	573	637	632	584	566	**526**	579	602	**579**

Note. The best performance of the column is highlighted in bold; SVM: support vector machine; kNN: k-nearest-neighbors; CART: classification and regression tree. ^a^: The lowest MATE value of ${\mathrm{MATE}}_{A,B}$; ^b^: The lowest MATE value of ${\mathrm{MATE}}_{B,C}$; ^c^: The lowest MATE value of ${\mathrm{MATE}}_{\mathrm{overall}}.$

## Data Availability

The data presented in this study are available on request from the corresponding author. The data are not publicly available due to privacy.
